# A Full View of Papillary Craniopharyngioma Based on Expanded Endonasal Approach: A Comprehensive Clinical Characterization of 101 Cases

**DOI:** 10.3390/jcm12206551

**Published:** 2023-10-16

**Authors:** Yanfei Jia, Kefan Cai, Ning Qiao, Fangzheng Liu, Wentao Wu, Siming Ru, Youchao Xiao, Lei Cao, Songbai Gui

**Affiliations:** Department of Neurosurgery, Beijing Tiantan Hospital, Capital Medical University, Beijing 100070, China; jiayanfei1988@163.com (Y.J.); 122021010893@mail.ccmu.edu.cn (K.C.); qiaoning@ccmu.edu.cn (N.Q.);

**Keywords:** craniopharyngioma, endoscopic surgery, papillary craniopharyngioma

## Abstract

Objective: The enlarged endonasal approach (EEA) has emerged as the preferred surgical procedure for removing craniopharyngiomas, due to its advantages of direct visualization and reduction of blind corners. However, owing to a low incidence of papillary CPs (PCPs) compared to adamantinomatous CPs (ACPs), a full view of PCP based on the EEA approach is limited. In this paper, the authors present the largest series to date analyzing the clinical characteristics based on the EEA approach for PCPs. Methods: A retrospective review was conducted on 101 PCPs patients who underwent endoscopic endonasal surgery (EEA) and whose condition was confirmed via postoperative pathology. The PCPs were classified into three types based on MRI data and intraoperative findings from EEA: suprasellar/intra-suprasellar (3V floor intact) type (Type I), suprasellar/intra-suprasellar (3V floor invasive) type (Type II), and intra-third ventricle type (Type III). The general characteristics of the three types of tumors were summarized, and postoperative follow-up was conducted to record detailed information on changes in vision, endocrine replacement, tumor recurrence, and quality of life. Results: Out of the 101 cases, 36 (36.64%) were classified as type I, 52 (51.49%) as type II, and 13 (12.87%) as type III. The mean age of type III patients was 40.46 ± 14.15 years old, younger than the other two types (*p* = 0.021). Headache (84.62%) and memory decline (61.54%) were prominent features in patients with type III (*p* = 0.029). Visual impairment was more common in type II (80.77%, *p* = 0.01). Gross total resection (GTR) was achieved in 91 patients (90.10%). There were no significant differences in GTR rates among the three types of tumors. There were significant differences in quality of life among the three types of PCP (*p* = 0.004), and type III presented with the highest rate of good postoperative quality of life (92.31%) based on the KPS score. Thirteen (12.87%) tumors recurred within a mean follow-up time of 38 (range, 8–63) months. Type II PCPs (OR 5.826, 95%CI 1.185–28.652, *p* = 0.030) and relapsed patients (OR 4.485, 95%CI 1.229–16.374, *p* = 0.023) were confirmed as independent risk factors for tumor recurrence. Conclusions: Most of the PCPs including intra-third ventricle PCPs can be safely and effectively removed through neuroendoscopy with EEA. Suprasellar/intra-suprasellar (third cerebral ventricle floor-invasive) type PCPs may have a worse postoperative quality of life compared to the other two types, and it may be a strong predictor of tumor recurrence.

## 1. Background

Craniopharyngioma (CP) is a rare epithelial tumor of the sella region derived from remnants of the Rathke’s pouch, and it is histologically low-grade (WHO grade I). Globally, CP constitutes 1.2–4.6% of all intracranial tumors, accounting for 0.5–2.5 new cases per 1 million people annually [1,2]. Two main histological subtypes of CP have been identified, namely adamantinomatous CP (ACP) and papillary CP (PCP), and they differ in developmental and histological features [3]. Crotty et al., observed that PCPs differed from ACPs in their lack of calcifications, predilection in involving the third ventricle, and almost exclusive occurrence in adulthood [4]. Moreover, infiltration of the adjacent brain tissue has not been identified in PCPs [4], a microscopic characteristic that has been linked to the presumed less aggressive behavior and low recurrence tendency [5,6]. Nevertheless, some surgical studies aimed at assessing differences in outcomes between PCPs and ACPs have reported contradictory results [6,7,8,9].

The expanded endonasal approach (EEA) provides in-line access and allows direct visualization with less blind corners. During CP resection through EEA, the pituitary stalk, hypothalamus, and the origin of CP can be definitively identified in almost all cases. EEA for CPs has been increasingly adopted given the above advantages [10,11,12,13,14,15].

The present study analyzed 101 patients undergoing endoscopic transnasal resection of PCPs, which is the largest PCP series ever documented based on the EEA. Our analysis includes sex, age, preoperative symptoms, hypophyseal hormone alterations, image characteristics, hypothalamic involvement, tumor origin, tumor resection rate, postoperative quality of life (Qol), etc. According to the MRI results combined with intraoperative findings from EEA, we classified PCPs into three types and we describe the characteristics of different types here. These findings may help offer further insight into PCPs and eventually facilitate the improvement in surgical treatment and outcomes for dealing with these challenging lesions.

## 2. Methods

Between September 2019 and December 2022, a total of 101 patients diagnosed with PCPs underwent EEA-based surgery at the Department of Neurosurgery, Beijing Tiantan Hospital, Capital Medical University. All individuals provided informed consent, which was duly authorized by the ethics committee of Beijing Tiantan Hospital of Capital Medical University. The ethical committee approval number was KY 2021-041-02.

### 2.1. Imaging Evaluation

Tumor volume, texture, and calcification were assessed via preoperative computed tomography (CT) and MRI scans. Tumor volumes were calculated using the following formula: volume = 4/3π (a/2 × b/2 × c/2), where a, b, and c represent the diameters in the three dimensions. CPs with hypothalamic adhesion or invasion can be evaluated based on the pre-operative Puget grading system [16]. Non-contrast CT scans were acquired within the first 6 h after surgery. Post-operative MRI scanning was performed within 2 weeks of surgery to confirm the extent of tumor removal. Gross total removal (GTR) was defined as resection without visible tumor remnants, according to intraoperative assessment and post-operative MRI data.

### 2.2. Surgical Planning and Techniques

A total of 101 patients diagnosed with PCP were included in the study, in which the EEA technique was employed. The main objective was to ensure complete removal of the tumor and minimize the chances of recurrence. In order to assess the availability of corridors for tumor resection, a sagittal MRI was conducted, specifically examining the nasal apex and the chiasm. This evaluation utilized the nasal chiasm line (NCL). The NCL effectively divided the tumors into two distinct regions, namely the upper supra-NCL region and the lower infra-NCL region. In cases where the supra-NCL region constituted more than 20% of the tumor, the lamina terminalis corridor was utilized to excise this portion of the tumor. Concurrently, if the tumor exceeded 10% of the infra-NCL region, both TLTA and TCPC techniques were employed. The step-by-step details of the surgical procedures can be found in previous publications [17,18,19]. An intraoperative visual evoked potential (VEP) was used to monitor visual function [20]. Finally, the skull base reconstruction was performed as described previously [17,20].

### 2.3. Classification of PCP Based on Preoperative Imaging Findings and EEA Approach

**Figure 1:**
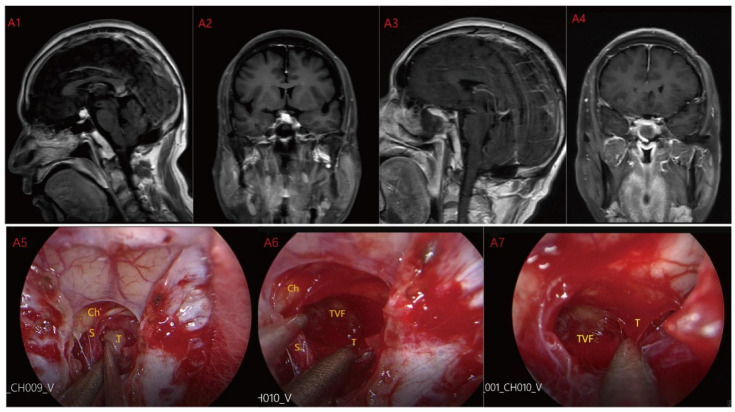
Case 1: Type I: Suprasellar/intra-suprasellar (3V floor intact) type. The third ventricular floor (TVF) and mammillary bodies (MB) were not involved preoperatively (**A1**,**A2**). The EEA approach was chosen for surgery, and the TCPCA was used. Intraoperative photographs (**A5**) show the pituitary stalk (PS) and surrounding perforating vessels. The tumor was located lateral to the PS, and the pituitary stalk was kept intact during the operation. After most of the tumor was resected, the complete third ventricle floor could be seen (**A6**,**A7**). Finally, the tumor was completely resected (**A3**,**A4**) and the third ventricle floor was completely preserved. Ch = optic chiasm TVF = third ventricle floor T = tumor S = pituitary stalk.

**Figure 2:**
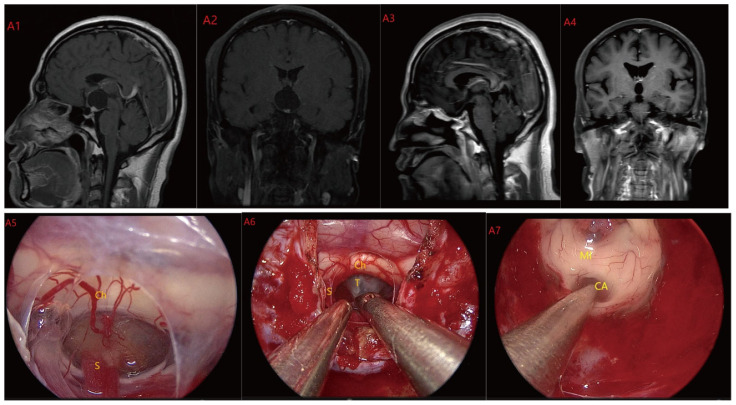
Case 2: Type II: The tumor located on the suprasellar region and compressed or penetrated the third ventricle floor (TVF) (**A1**,**A2**). During EEA surgery, the complete pituitary stalk can be seen via TCPCA, and the tumor is located behind and above the pituitary stalk (**A5**,**A6**). The tumor adhered closely to the floor of the third ventricle, and part of it protruded into the third ventricle during the operation. The tumor achieved GTR (**A7**). Postoperative MRI were shown in (**A3**,**A4**). Optic chiasm (Ch), foramen of Monroe, massa intermedia (MI), cerebral aqueduct (CA), tumor (T), pituitary stalk (S).

**Figure 3:**
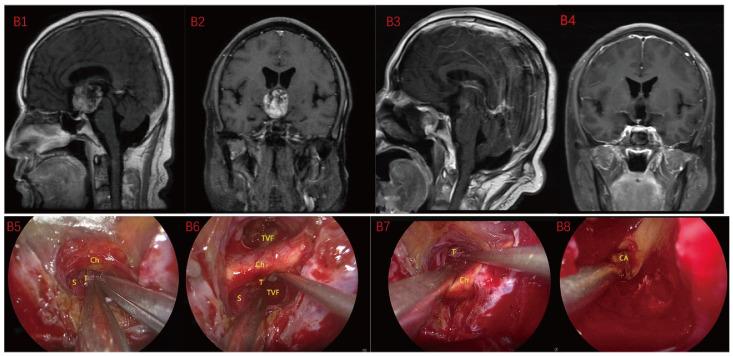
Case 3 Type II: (**B1**,**B2**) The tumor was located on the suprasellar, growing upwards into the third ventricle, and it is impossible to judge whether the third ventricle floor was complete before operation. During the EEA surgery (**B5**–**B8**), the TCPCA and TLTA was used. The tumor was completely resected (**B3**,**B4**), and the floor of the third ventricle was seen to be incomplete. Optic chiasm (Ch), foramen of Monroe, massa intermedia (MI), cerebral aqueduct (CA), tumor (T).

**Figure 4:**
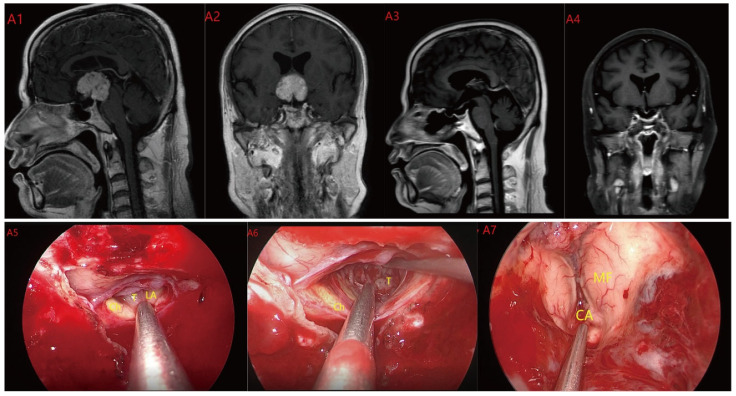
Case 4 Type III: the tumor grows strictly inside the third ventricle. (**A1**,**A2**) The TLTA was used; the pituitary stalk could not be seen during the surgery (**A5**–**A7**). Finally, the tumor was totally resected (**A3**,**A4**). Optic chiasm (Ch), foramen of Monroe (MF), massa intermedia (MI), cerebral aqueduct (CA), tumor (T).

**Figure 5:**
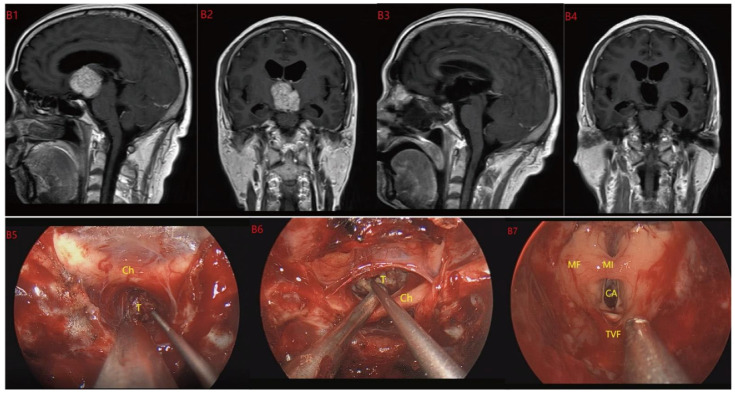
Case 5 Type III: the tumor grows strictly inside the third ventricle. (**B1**,**B2**) The TCPCA AND TLTA was used; the pituitary stalk could not be seen during the surgery (**B5**–**B7**). The TVF was intact. The tumor was finally totally resection (**B3**,**B4**). Optic chiasm (Ch), foramen of Monroe (MF), massa intermedia (MI), cerebral aqueduct (CA), tumor (T).

Based on MRI data and intraoperative findings from EEA, we classified PCPs into three types: (1) Type I: Suprasellar/intra-suprasellar (3V floor intact) type: most tumors originate from the segments of the pituitary stalk between diaphragma and infundibulum, as shown in Figure 1. (2) Type II: Suprasellar/intra-suprasellar (3V floor invasive) type: most of the tumors originate from pituitary stalk (PS), or infundibulum and median eminence (ME); the interface between tumor and 3V floor is not clear or has disappeared, and the tumor infiltrates into the bottom of the third ventricle, as shown in Figure 2 and Figure 3. (3) Type III: Intra-third ventricle type; most of the tumors originate from the infundibulum and ME, the tumor grows strictly inside the third ventricle, the 3V floor is intact and covered with arachnoid membrane, as shown in Figure 4 and Figure 5.

### 2.4. The Extent of Tumor Resection

A volumetric analysis of MRI images was performed pre- and postoperatively to determine the extent of tumor resection. The term gross total resection (GTR) signifies the elimination of the entire tumor, while the term subtotal resection (STR) signifies the removal of 90% or more of the tumor, and the term partial resection (PTR) signifies the removal of less than 90% but more than 50% of the tumor. MRI was used to detect tumor recurrence during the follow-up period by demonstrating the appearance of new pathological tissue or growth of tumor remnants. Follow-up MRI scanning was performed 1 week and 3 months after the surgery and at regular intervals of 6–12 months.

### 2.5. Endocrine Status

The pre- and post-operative assessment of patients included evaluation of the endocrine status, primarily focusing on their adenohypophysis function and diabetes insipidus. To assess adenohypophysis function, comprehensive serum pituitary hormone panels were utilized. Diabetes insipidus (DI) was defined as a urine volume exceeding 50 mL/kg/d. Electrocyte imbalance was defined as a serum sodium level surpassing 145 mmol/L or dipping below 135 mmol/L, specifically observed within two weeks after the surgical procedure. The Body Mass Index (BMI) was evaluated for all patients both at the time of surgery and during their final visit. Obesity was classified as a preoperative BMI exceeding 30 kg/m^2^ and a subsequent 9% increase in BMI after surgery, in comparison to the pre-operative BMI.

### 2.6. Statistical Analysis

The SPSS software (version 25, IBM Corp., Armonk, NY, USA) was utilized to conduct statistical analyses. Descriptive statistics were employed to depict the sociodemographic characteristics of the participants, as well as their tumor characteristics and factors related to treatment. For data that followed a normal distribution with equal variances, a *t*-test was performed to analyze categorical variables between two groups. Associations or proportions were assessed using a chi-square test and Fisher’s exact test. Any *p*-value below 0.05 was considered to be statistically significant. Mean ± SD or median (IQR) were used to present normally distributed or non-normally distributed samples, respectively. To compare continuous variables between groups, the Mann–Whitney U-test was employed. The correlation between risk factors of tumor recurrence was examined through binary logistic regression. Variables with a *p*-value below 0.1 were included in multivariate binary logistic regression analysis. Tumor recurrence was considered the dependent variable, and a multiple regression analysis was conducted, leading to the establishment of the regression equation.

## 3. Results

### 3.1. Baseline Patient Characteristics

Baseline patient characteristics among different groups are summarized in Table 1.

The mean age of 101 patients at diagnosis was 48.54 ± 12.99. The mean age at diagnosis of the 101 patients was 48.54 ± 12.99, while the mean age of type III was 40.46 ± 14.15, significantly younger than the other two tumor types (*p* = 0.021). Headache (84.62%) and memory decline (61.54%) are prominent features of patients with type III compared with the other two types (*p* = 0.029). Visual impairment is more common in type II (80.77%, *p* = 0.01). Although not statistically different, type III tumors were relatively less prone to preoperative hypopituitarism compared with type I and type II.

### 3.2. PCP Characteristics Based on Pre-Operative Imaging and Intraoperative Findings

Preoperative MRI characteristics and intraoperative findings among different groups using EEA are summarized in Table 2. The mean tumor volumes of the three types were statistically different (*p* < 0.001). We found that the tumor volume of type III was 12.64 ± 6.27, which was larger than that of the other two types. Obviously, type III was more likely to combine with obstructive hydrocephalus (*p* < 0.001) and peritumoral edema (*p* = 0.016). Overall, 89 of the 101 (88.12%) tumors were regular in shape and cystic solid tumors were the most common (47.52%).

Intraoperatively, we found tumor origins differed significantly among the three tumor types (*p* < 0.001). We believe that all of Type III and half of Type II originate from Infundibulum and ME. The three types of tumors differed significantly in the choice of surgical approach (*p* < 0.001). TCPCA was used in all type I tumors, and in type II, 46 (88.46%) cases used TCPCA, 6 (11.54) cases used TCPCA AND TLTA. In type III, three (23.08%) cases used TCPCA, seven (53.85%) cases used TLTA, and three (23.08%) cases used TCPCA and TLTA. In total, 91 of 101 (90.01%) achieved GTR and the rate of GTR showed no significant difference among the three groups.

### 3.3. PCP Characteristics Based on Post-Operative Findings and Follow-Up

PCP characteristics based on postoperative and follow-up data of different groups are summarized in Table 3. Of the 68 patients with preoperative visual impairment, 48 (70.58%) had better vision than after surgery. Electrolyte disturbances occurred in 41.58% of patients. There are significant differences in the incidence of postoperative DI in three types of tumors (*p* = 0.05). Within a mean follow-up time of 38 (range, 8–63) months, 13 patients exhibited tumor recurrence (12.87%); 11 of them were type II, and there was 1 case of type I and 1 case of type III.

Additionally, there were significant differences in quality of life after three types of PCP (*p* = 0.004), Type III had the highest rate of good postoperative quality of life (92.31%) with a KPS score > 70.

### 3.4. Multivariate Analysis of Tumor Recurrence: A Stepwise Multivariate Logistic Regression Analysis for Risk Factors of Tumor Recurrence

**Figure 6:**
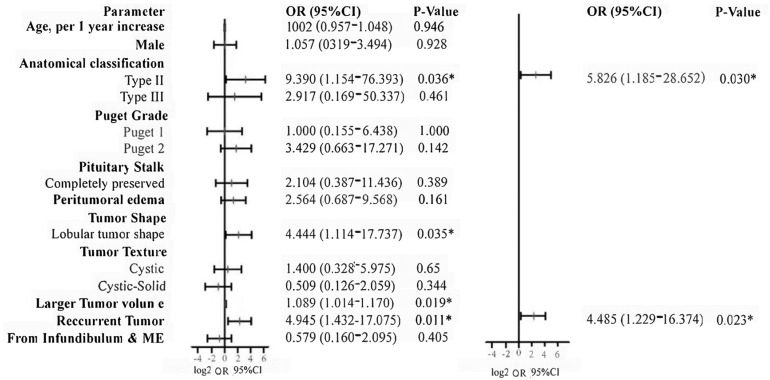
Binary logistic regression analysis was used to identify the risk factors of tumor recurrence. According to the analysis, type II tumors and recurrent tumors were confirmed as independent risk factors for tumor recurrence. *: *p* value < 0.05.

We included the factors that may cause tumor recurrence, including tumor volume, Puget grade, anatomical type, texture, and surgical approach, tumor origin, etc. We used tumor recurrence as the dependent variable. Next, the above variables were included in the multivariate logistic regression (stepwise method), and finally, the Type II PCPs (OR 5.826, 95%CI 1.185–28.652, *p* = 0.030) and relapsed patients (OR 4.485, 95%CI 1.229–16.374, *p* = 0.023) were confirmed as independent risk factors of tumor recurrence, as shown in Figure 6.

## 4. Discussion

Due to the limited and scattered information available in the medical literature, comprehending the clinical features of PCPs can be challenging. To address this issue, past neurosurgeons have categorized CPs into six distinct types, based on pre-operative imaging and light microscopy findings during surgery. These classifications are as follows: (a): purely intrasellar-infradiaphragmatic; (b): intra-and suprasellar, infra- and supradiaphragmatic; (c): supradiaphragmatic, para-chiasmatic, extra-ventricular; (d): intra- and extraventricular; (e): paraventricular with respect to the third ventricle; and (f): purely intraventricular [21,22]. It is well known that the tumor and surrounding vital structures are sometimes unclear, with incorrect MRI definitions being reported frequently even by experienced doctors**.** In addition, light microscopy is always unable to provide a full view of the tumor and surrounding structures due to obstruction of peripheral normal tissue and blind spots. Due to this, the classification based on microsurgery is unable to clarify the origin and growth pattern of tumors, which is crucial information when planning appropriate surgical approaches. For the first time, we classified PCPs into three types based on MRI combined with intraoperative findings from EEA, and described the characteristics of different types as follows.

### 4.1. Baseline Characteristics of Different Types of PCPs

In contrast to the characteristic bimodal age (first peak in children) of ACPs, PCP occurs almost exclusively in adults with a mean patient age of 40–55 years [4]. There was no patient under the age of 18 in our cohort, and the mean age at the presentation of PCP was 40.54 ± 12.99 years. Interesting enough, the average age of Type III PCPs was 40.46 ± 14.15 years, which was obviously lower than other two types (*p* < 0.05). It was reported that ciliated or goblet cells were observed at focal areas of columnar epithelium in PCPs histopathology, and patients with ciliated or goblet PCPs were a decade younger [6]. In our series, however, there was no significant difference in pathological results in different PCPs types. We believe that Type II and Type III PCPs tend to interfere with the cerebrospinal fluid (CSF) circulation, often leading to an obstructive hydrocephalus (headaches and memory impairment), which can be diagnosed at an early stage. Contrary to the male predominance in several reports [4,6], we found that 39.60% of PCP patients were males, a rate slightly lower than that of females (60.40%) but without statistically significant difference (*p* > 0.05).

In this study, headache (52.48%), visual impairment (67.33%), hypomnesia (32.67%), diabetes and insipidus (36.63%) were the most common signs among all preoperative symptoms. The BMI of our patients was 25.32 ± 4.17, and there was no difference between these groups with respect to BMI (*p* > 0.05). Type III PCPs patients suffered more headache (84.62%) and hypomnesia (61.54%), compared with Type I and Type II PCPs patients (*p* < 0.05). The mean tumor volume was 12.64 ± 6.27 cm^3^ and hydrocephalus existed in 46.15% of the Type III PCPs patients; these are all significantly higher numbers than those in Type I and Type II PCPs patients (*p* < 0.01). Clinical symptoms were associated with the location of the lesion. Type III PCPs arise in the third ventricle and often lead to the blockage of CSF circulation. Hence, this type of PCPs patient was more likely to display headache and hypomnesia which indicated increased intracranial hypertension.

Compared with Type III PCPs, the positions of Type I and Type II PCPs are more adjacent to the visual pathway and pituitary gland. Our study showed that the incidence of visual impairment for Type I, Type II and Type III was 55.56%, 80.77% and 46.15%, respectively (*p* < 0.05). Although there was no statistical significance, it should be noted that the incidences of hypoadrenalism, hypothyroidism, and hypogonadism was highest in Type II, lower in Type I, and the lowest in Type III PCPs (*p* > 0.05). All these results indicated that both Type I and Type II PCPs patients were more susceptible to visual impairment and hypopituitarism symptoms compared to Type III PCPs.

### 4.2. Imaging Characteristics and Intraoperative Findings of Different Types of PCPs

In contrast to the mixed–solid consistency and lobulated morphology of the ACPs, solid consistency and round shape were the major morphological characteristics of the papillary type [23]. Further research showed that PCPs tumor consistencies were solid (50%) or cystic with a cauliflower-like solid nodule inside (18%), and 72% of the PCPs had a round smooth shape [6]. In this study, all PCPs texture were classified as follows: cystic lesions (19.80%), solid lesions (32.67%), cystic–solid lesions (47.52%). Further analysis revealed that 55.56% of the tumors were cystic–solid lesions in Type I PCPs, which was significantly higher than Type II and Type III PCPs (*p* < 0.05). In addition, we found that 61.54% of tumors were solid lesions in Type III PCPs, which was significantly higher than Type II (36.54%) and Type I (16.67%) PCPs (*p* < 0.05). Previous research implied that low-risk adhesions predominate among solid lesions, which may be associated with a high total surgical removal rate [6]. However, there was no significant difference in the resection rate between the cystic lesions and solid lesions in this study. Our research suggests that tumor texture may be irrelevant to the adhesion and resection of the PCP through EEA.

In this study, we found that 30.77% of the tumors showed peritumoral edema in the Type III PCPs, which was significantly higher than in Type II (23.08%) and Type I (2.78%) PCPs (*p* < 0.05). Peritumoral edema is an important semantic feature when predicting brain invasion of cerebral tumor, which was consistently reported by previous studies [24,25]. Meanwhile, Puget grading 2 PCPs accounted for the highest proportion in Type III PCPs (69.23%), Type II PCPs (51.92%) accounted for the second highest, and Type I PCPs (0.00%) accounted for the lowest (*p* < 0.05). The results suggested that Type III PCPs are the most invasive and aggressive with regard to their involvement of the hypothalamus among three subtypes.

Since CPs grow along with the longitudinal axis of the pituitary stalk, the EEA allows direct access to the long axis of the tumor, a factor well recognized as critical to defining the origin of a tumor. During the operation, 21.78% of the PCPs tumors originated from the PS, and 78.22% of the PCPs tumors originated from the infundibulum and median eminence (ME) in this study. In particular, all the Type III PCPs originated from the infundibulum and median eminence (ME). Squamous metaplasia of adenohypophyseal cells in the pars tuberalis has been considered to cause PCPs [26]. However, most PCPs originated from the Infundibulum and ME in our cohort, which supports that the interaction between Rathke’s pouch remnants and 3V nervous tissue played an embryological role in PCP formation [27].

In our cohort, 91.09% of the tumors’ PS were partially or totally preserved, and the PS preservation in Type II PCPs was 84.62% which was lower than those in other groups but without statistically significant difference (*p* > 0.05). During the surgical process of Type I and Type III PCPs via EEA, the PS was easy to distinguish and protect from tumor tissue. Instead, part of the PS in Type II PCPs was exhibited as a trans-pituitary stalk–funnel growth trend. In this case, PS always fused with the tumor and presented as a membranous structure, and it was difficult to distinguish and protect PS from tumor tissue.

### 4.3. Treatment and Surgical Approach

Undoubtedly, total surgical resection is the best treatment for CP [6,28,29,30,31]. Compared to the conventional transcranial approach used to remove CPs, the endoscopic transsphenoidal approach achieves better GTR with less residual tumor, effectively reducing morbidity and mortality [15,28,31,32,33]. Two surgical corridors (trans-chiasm-pituitary and trans-lamina terminalis) were used to resect CPs through EEA [17,19,34]. In Kitano’s study, 20 suprasellar cases with CP underwent extended transsphenoidal surgery via infra-chasmatic access, combined with or without a suprachiasmatic trans-lamina terminalis approach [19]. Furthermore, 82 cases of third ventricle tumors were resected by EEA in Seo’s article [34]. It provides direct visualization during surgery for dissecting tumor interfaces, pituitary stalk, and hypothalamus, maintaining their blood supply and functional integrity, as well as preventing complications associated with extensive craniotomies and brain retraction.

In this cohort, all of the Type I PCPs were treated via EEA through TCPCA; 88.46% of the Type II PCPs underwent TCPCA and 11.54% underwent TCPCA AND TLTA; the incidence of TCPCA, TLTA and TCPCA AND TLTA were 23.08%, 53.85% and 23.08% used for Type III PCPs, respectively (*p* < 0.05). Most of the time, the sub-chiasmic space was dissected to remove the tumors. If the infra-chiasmic corridor was too narrow, a supra-chiasmic corridor would be used, especially in Type III PCPs. It was reported that trans-lamina terminalis corridor is a minimally invasive corridor used in EEEA to treat intrinsic third ventricular craniopharyngioma [35]. For seven Type III PCPs, the tumors compressed the chiasm inferiorly and the IC was very narrow; therefore, the suprachiasmatic trans-lamina terminalis corridor between the chiasm and the anterior communicating artery was used. For six Type II PCPs and three Type III PCPs, the tumors were distributed in the vertical axis and optic chiasm located in the middle of the surgical field, and they were resected through TCPCA AND TLTA.

Compared to ACPs, PCPs have been deemed more suitable for total removal because they do not infiltrate the hypothalamus tissue as much. This may be due to Adamson et al.’s hypothesis that PCPs have a smooth interface with the brain, without the typical finger-like protrusions found in ACPs, which contribute to their higher resection rates [5]. However, whether endoscopic or completed via microscopy, the rate of gross total removal was only 36–58% in a systematic review comprising 239 PCP patients [4,6]. In our cohort, the overall tumor total resection rate was 90.10%, the GTR in Type II PCPs was 84.62% which was lower than those in the other two groups but without statistically significant difference (*p* > 0.05). During the operation, neither critical blood vessels nor neural structures were damaged in our cohort. Again, our results showed that EEA could effectively control PCP tumors and their surrounding normal tissues.

### 4.4. Postoperative Complications and Qol

The complications following CP excision through EEA included diabetes insipidus (DI), electrolyte disorder, CSF, intracranial infection, and pituitary hormone deficiencies [10,11,18,33,36,37]. Previous studies have shown that post-operative DI occurrence ranges from 7.5% to 54.2% in CP patients [36,37]. They also reported that 40.8% CPs patients experienced a weight gain of 35% or higher within the first year of surgery, with an average weight gain of 17.59 ± 12.28% [38,39]. Pituitary hormone deficiencies have been reported in 54–100% of CP patients, with post-operative ACTH deficiency occurring in 55–88% of patients, GH deficiency in 88–100% of patients, TSH deficiency in 39–95% of patients, gonadotropin deficiency in 80–95% of patients, and arginine and vasopressin deficiency in 25–86% of patients [31,40].

In this study, 19.80% patients had visual impairment; 47.52% patients had diabetes insipidus; 41.58% patients had hyponatremia; 6.93% patients had hypomnesia; 3.96% patients had CSF; 6.93% patients had intracranial infections, and 36.63% patients were obese, and there was no significant difference among the three groups (*p* > 0.05). Detection of hypophyseal hormones showed that 63.37%, 10.89%, 23.76%, 70.30%, and 38.61% patients had ACTH, T3, GH, T, and E2 deficiencies, respectively; however, there was no significant difference among the three groups (*p* > 0.05). Significantly, the preoperative incidence of patients with hypomnesia fell from 61.54% to 15.38% after operation in Type III PCPs. This result indicates that EEA can effectively improve the patients’ consciousness disorder in Type III PCPs. Notably, the quality-of-life scores for patients with Type II PCPs were significantly reduced compared with Type I and Type III PCPs (*p* < 0.05), meaning that Type II PCPs may be relevant to poor prognosis.

Previous studies showed that the PCP recurrence rate was 7–40%, which was not significantly lower than the recurrence rates found in the case of ACPs [6,41,42,43,44,45]. In our cohort, the overall tumor recurrence rate was 12.87%; the incidence of tumor recurrence was 2.78%, 21.15%, and 7.69% in Type I, Type II, and Type III PCPs, respectively (*p* < 0.05). It implied that Type II PCPs were more likely to recur. Through univariate and multiple logistic regression analyses, Type II PCPs and patients with relapse were directly associated with tumor recurrence. Due to tumor invasion of the floor of the third ventricle, there were longer and wider tumor margins in Type II PCPs. Meanwhile, after undergoing one or multiple surgeries, the arachnoid interface between tumor and normal surrounding tissue is eliminated and replaced with inflammatory hyperplasia tissue. The above two circumstances will increase the difficulty of surgery and reduce the resection rate.

## 5. Conclusions

Based on MRI combined with intraoperative findings from EEA, we classified PCPs into three types and described the characteristics of different types. Moreover, we found that both Type I and Type II PCPs were more prone to visual impairment and hypopituitarism symptoms; Type III PCPs were more likely to lead to headaches and hypomnesia. Furthermore, Type III PCPs were found to have the largest tumor volume, the lowest onset age, and the highest proportion of solid tumor, and all the Type III PCPs originated from the infundibulum and ME. In addition, most of the PCPs, including Type III PCPs, can be safely and effectively removed through neuroendoscopic treatment with EEA. Finally, it was implied that the suprasellar/intra-suprasellar (3V floor invasive)-type PCPs may be relevant to the slightly poor prognosis observed in this study.

This study also has several limitations. The sample size is relatively small, although it is the largest series of PCP patients undergoing EEA reported to date. Second, the follow-up duration was short, with some patients still in the follow-up period. Furthermore, this study was a retrospective study, and there may have been selection bias during retrospective data collection.

## Figures and Tables

**Table 1:** Clinical presentation of patients with different types of PCP.

Parameter		Type I	Type II	Type III	Total	χ2/F	*p*
	Number of Patients	N = 36 (Case, %)	N = 52 (Case, %)	N = 13 (Case, %)	N = 101 (Case, %)		
**Age**		47.53 ± 13.94	51.27 ± 11.19	40.46 ± 14.15	40.54 ± 12.99	4.000	0.021 *
**Gender**	**Male**	18 (50.00)	19 (36.54)	3 (23.08)	61 (60.40)	3.315	0.191
	**Female**	18 (50.00)	33 (63.46)	10 (76.92)	40 (39.60)		
**Secondary operation**		6 (16.67)	12 (23.08)	1 (7.69)	19 (18.81)	1.780	0.411
**Headache**		15 (41.67)	27 (51.92)	11 (84.62)	53 (52.48)	7.078	0.029 *
**Visual impairment**		20 (55.56)	42 (80.77)	6 (46.15)	68 (67.33)	9.188	0.010 *
**Hypomnesia**		3 (8.33)	22 (42.31)	8 (61.54)	33 (32.67)	16.813	0.000 **
**DI**		15 (41.67)	21 (40.38)	1 (7.69)	37 (36.63)	5.399	0.067
**Pre-BMI**		24.24 ± 3.70	25.85 ± 4.52	26.20 ± 3.59	25.32 ± 4.17	1.934	0.15
**Hypoadrenalism**	**ACTH**	10 (27.78)	6 (11.54)	1 (7.69)	17 (16.83)	4.898	0.086
**Hypothyroidism**	**T3**	4 (11.11)	2 (3.85)	0 (0.00)	6 (5.94)	2.952	0.229
**Hyposomatotropism**	**GH**	7 (19.44)	17 (32.69)	3 (23.08)	27 (26.73)	2.008	0.366
**Hypogonadism**	**T**	25 (69.44)	27 (51.92)	5 (38.46)	57 (56.44)	4.617	0.099
	**E2**	19 (52.78)	21 (40.38)	5 (38.46)	45 (44.55)	1.547	0.461

DI: Diabetes insipidus; BMI: body mass index; ACTH: adrenocorticotropic hormone; T3: Triiodothyronine; GH: growth hormone; T: testosterone; E2: estradiol. *: *p* value < 0.05, **: *p* value < 0.01.

**Table 2:** PCP characteristics based on imaging findings and EEA approach.

Parameter		Type I	Type II	Type III	Total	χ2/F	*p*
	Number of Patients	N = 36 (Case, %)	N = 52 (Case, %)	N = 13 (Case, %)	N = 101 (Case, %)		
**Tumor volume**		4.07 ± 4.60	9.66 ± 7.30	12.64 ± 6.27	8.05 ± 7.01	12.223	0.000 **
**Hydrocephalus**		0 (0.00)	14 (26.92)	6 (46.15)	20 (19.80)	16.234	0.000 **
**Tumor texture**	**Cystic lesions**	10 (27.78)	9 (17.31)	1 (7.69)	20 (19.80)		
	**Solid lesions**	6 (16.67)	19 (36.54)	8 (61.54)	33 (32.67)	9.936	0.042 *
	**Cystic–solid lesions**	20 (55.56)	24 (46.15)	4 (30.77)	48 (47.52)		
**Tumor shape**	**Round circular**	35 (97.22)	42 (80.77)	12 (92.31)	89 (88.12)	5.57	0.056
	**Lobular**	1 (2.78)	10 (19.23)	1 (7.69)	12 (11.88)		
**Peritumoral edema**		1 (2.78)	12 (23.08)	4 (30.77)	17 (16.83)	8.832	0.016 *
**Puget grading**	**Puget 0**	17 (47.22)	8 (15.38)	1 (7.69)	26 (25.74)		
	**Puget 1**	19 (52.78)	17 (32.69)	3 (23.08)	39 (38.61)	34.232	0.000 **
	**Puget 2**	0 (0.00)	27 (51.92)	9 (69.23)	36 (35.64)		
**PS preservation**		35 (97.22)	44 (84.62)	13 (100.00)	92 (91.09)	5.652	0.060
**Tumor origin**	**PS**	15 (41.67)	7 (50.00)	0 (0.00)	22 (21.78)		
	**Infundibulum and ME**	21 (58.33)	7 (50.00)	13 (100.00)	79 (78.22)	14.088	0.001 **
**Surgical approach**	**TCPCA**	36 (100.0)	46 (88.46)	3 (23.08)	85 (84.16)		
	**TLTA**	0 (0.00)	0 (0.00)	7 (53.85)	7 (6.93)	60.874	0.000 **
	**TCPCA AND TLTA**	0 (0.00)	6 (11.54)	3 (23.08)	9 (8.91)		
**Extent of resection**	**GTR**	35 (97.22)	44 (84.62)	12 (92.31)	91 (90.10)	3.872	0.144
	**STR**	1 (2.78)	8 (15.38)	1 (7.69)	10 (9.90)		

PS: pituitary stalk; ME: median eminence; TCPCA: trans-chiasm-pituitary corridors approach; TLTA: trans-lamina terminalis approach; GTR: gross total resection; STR: subtotal total resection. *: *p* value < 0.05, **: *p* value < 0.01.

**Table 3:** Postoperative clinical information of different types of PCPs.

Parameter		Type I	Type II	Type III	Total	χ2	*p*
	Number of Patients	N = 36 (Case, %)	N = 52 (Case, %)	N = 13 (Case, %)	N = 101 (Case, %)		
**Visual impairment**		7 (19.44)	11 (21.15)	2 (15.38)	20 (19.80)	0.222	0.895
**DI**		23 (63.89)	20 (38.46)	5 (38.46)	48 (47.52)	6.007	0.050 *
**Electrolyte disorder**	**Hyponatremia**	16 (44.44)	20 (38.46)	6 (46.15)	42 (41.58)	0.442	0.802
**Hypomnesia**		8 (22.22)	8 (15.38)	2 (15.38)	18 (17.82)	0.740	0.691
**CSF**		0 (0.00)	4 (7.69)	2 (15.38)	6 (5.94)	4.634	0.099
**Intracranial infection**		0 (0.00)	5 (9.62)	2 (15.38)	7 (6.93)	4.702	0.095
**Obesity**		14 (38.89)	18 (34.62)	5 (38.46)	37 (36.63)	0.189	0.910
**Hypoadrenalism**	**ACTH**	21 (58.33)	35 (67.31)	8 (61.54)	64 (63.37)	0.76	0.684
**Hypothyroidism**	**T3**	3 (8.33)	6 (11.54)	2 (15.38)	11 (10.89)	0.536	0.765
**Hyposomatotropism**	**GH**	4 (11.11)	15 (28.85)	5 (38.46)	24 (23.76)	5.473	0.065
**Hypogonadism**	**T**	26 (72.22)	34 (65.38)	11 (84.62)	71 (70.30)	1.941	0.379
	**E2**	18 (51.43)	18 (34.62)	3 (23.08)	39 (38.61)	4.078	0.13
**KPS ≥ 70**		35 (97.22)	37 (71.15)	12 (92.31)	84 (83.17)	11.217	0.004 **
**Tumor recurrence**		1 (2.78)	11 (21.15)	1 (7.69)	13 (12.87)	6.762	0.034 *

DI: Diabetes insipidus; CSF: cerebrospinal fluid; ACTH: adrenocorticotropic hormone; T3: Triiodothyronine; GH: growth hormone; T: testosterone; E2: estradiol; KPS: Karnofsky performance score. *: *p* value < 0.05, **: *p* value < 0.01.

## Data Availability

The data presented in this study are available on request from the corresponding author. To protect patient privacy, individual data cannot be made publicly available.

## Abbreviations

PCP: papillary CPs; EEA: enlarged endonasal approach; TVF: third ventricle floor; TCPCA: trans-chiasm-pituitary corridors approach; TLTA: trans-lamina terminalis approach.
